# Human Exposure Pathways of Heavy Metals in a Lead-Zinc Mining Area, Jiangsu Province, China

**DOI:** 10.1371/journal.pone.0046793

**Published:** 2012-11-13

**Authors:** Chang-Sheng Qu, Zong-Wei Ma, Jin Yang, Yang Liu, Jun Bi, Lei Huang

**Affiliations:** 1 State Key Laboratory of Pollution Control and Resource Reuse, School of the Environment, Nanjing University, Nanjing, China; 2 Department of Environmental Science & Engineering, Fudan University, Shanghai, China; 3 Department of Environmental Health, Rollins School of Public Health, Emory University, Atlanta, Georgia, United States of America; 4 Jiangsu Provincial Academy of Environmental Science, Nanjing, China; Stony Brook University, Graduate Program in Public Health, United States of America

## Abstract

Heavy metal pollution is becoming a serious issue in developing countries such as China, and the public is increasingly aware of its adverse health impacts in recent years. We assessed the potential health risks in a lead-zinc mining area and attempted to identify the key exposure pathways. We evaluated the spatial distributions of personal exposure using indigenous exposure factors and field monitoring results of water, soil, food, and indoor and outdoor air samples. The risks posed by 10 metals and the contribution of inhalation, ingestion and dermal contact pathways to these risks were estimated. Human hair samples were also analyzed to indicate the exposure level in the human body. Our results show that heavy metal pollution may pose high potential health risks to local residents, especially in the village closest to the mine (V1), mainly due to Pb, Cd and Hg. Correspondingly, the residents in V1 had higher Pb (8.14 mg/kg) levels in hair than those in the other two villages. Most of the estimated risks came from soil, the intake of self-produced vegetables and indoor air inhalation. This study highlights the importance of site-specific multipathway health risk assessments in studying heavy-metal exposures in China.

## Introduction

Heavy metals are known to be persistent in the human body, with excretion half-lives that last for decades. Heavy metals can lead to a wide range of toxic effects, such as carcinogenicity, mutagenicity and teratogenicity [1], [2], [3], [4]. Heavy metal contamination is a major environmental concern on a global scale, particularly in China, with its rapid economic development [5], [6], [7]. Due to large-scale production and consumption and lack of regulations, heavy metals such as lead (Pb), zinc (Zn), cadmium (Cd), mercury (Hg), and chromium (Cr) are emitted into the environment in large quantities through wastewater irrigation, solid waste disposal, sludge application, vehicular exhaust and atmospheric deposition [8]. As a result, heavy metals are present in industrial, municipal and urban runoff, and they continuously accumulate in the environment in China [9], [10], [11], [12], [13]. Since 2005, health related incidents caused by heavy metal pollution have risen sharply in China, with major accidents attracting nationwide attention [14].

Heavy metals have been found widely in various environmental media (including soil, water, air and food) around the world [5], [15], [16]. These species may enter the human body through inhalation of dust, direct ingestion of soil and water, dermal contact of contaminated soil and water, and consumption of vegetables grown in contaminated fields. Various studies have been conducted to evaluate population health risks due to heavy metal exposure through various exposure pathways, especially soil and food chain [17], [18], [19], [20], [21], [22]. Since it is difficult to identify the key exposure route because of lack of multipathway risk analysis, media or pathway-specific approach may fail to ensure public safety [23]. Therefore, it is necessary to assess the aggregate exposure to metals concerning about different environmental media and pathways. Previous studies have also demonstrated the importance of conducting multipathway risk assessment to identify the dominant pathway of potential concern [24], [25], [26], [27].

Human health risk assessment, as formalized in 1983 [28], has been recognized as an important tool for estimating the nature and probability of adverse health effects in humans who may be exposed to chemicals and for presenting risk information to the decision maker. The US Environmental Protection Agency (EPA) hazard quotients (HQ) are widely used to characterize non-carcinogenic health effects posed by heavy metals by comparing the exposure level with a reference dose [15], [22]. However, risk assessment is a complex process that is inherently linked with uncertainty [29]. Monte Carlo simulation is commonly used to quantify uncertainty in human health risk estimates [22]. It is a probabilistic approach that works with probability distributions rather than deterministic values of each parameter to estimate a risk.

For metal toxicity monitoring and environmental risk assessment, the identification of heavy metals from biological samples such as blood, urine or hair is useful for identifying exposure. Because the sampling is less invasive, more convenient to store and transport, and less hazardous to handle, hair has been used widely in biomonitoring environmental and occupational exposures of various pollutants [30], [31], [32], [33], [34]. Furthermore, hair sample can be a useful assessment tool in characterizing long-term exposure of the measured contaminant, whereas blood and urine often reflect most recent exposures. Hair has been used in biomonitoring of heavy metals on large cohorts in Brazil [35], determining geological source and exposure through fish consumption in Lake Victoria [36], characterizing human exposure in an abandoned mine in Portugal [32] and examining the residential exposure in an e-waste recycling area in southeastern China [31]. We focus our study on using metal content in hair as a biomonitoring indicator to identify whether differences of long-term exposure existed among residents in different villages.

The current study considered human exposure to heavy metals via drinking water, dietary intake, dermal contact and inhalation in the populations living near a lead-zinc mining area in Jiangsu Province, China. In addition to Pb and Zn, eight other major associated elements were included in this study: Ag (silver), Cd, Cr, Cu (copper), Ni (nickel), Se (selenium), Tl (thallium) and Hg (mercury). These ten metals are all priority pollutant metals (PP metals) as set by the USEPA. The aims of this study are: (1) to evaluate the potential health impacts of these metals on the general population in the mining area, (2) to provide a better understanding of each exposure pathway and (3) to help local governments to prioritize pollution control and health intervention policies to protect local population.

## Materials and Methods

### Ethics Statement

This study was approved by the review board of Nanjing University. All participants were informed about the objectives and methods of the study before the investigation. And written consent was obtained from all participants.

### Study Areas

**Figure 1:**
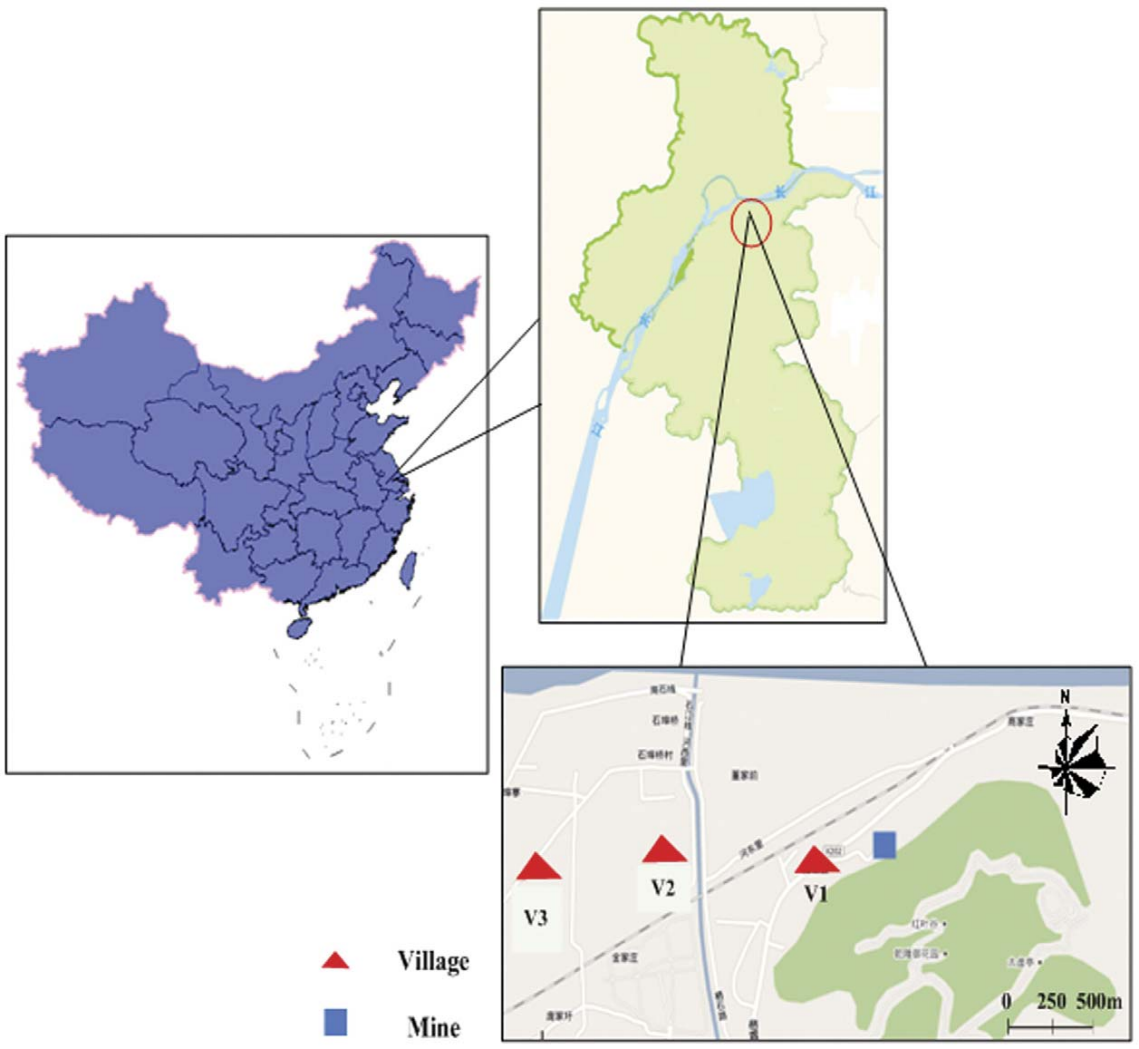
Locations of the study areas.

The studied Qixia lead-zinc mining area, with reserves of four million tons of lead and zinc, is an important mining region in Jiangsu province, which is one of the fastest-developing provinces in China. The mine has been excavated for 60 years, and the nearby area is suffering serious environmental deterioration from the wastewater and solid waste discharge. Three nearby villages at the leeward side of the mine were sampled in this study to identify the potential health risks and its sources. Village 1 (V1) is 100 m from the mine, village 2 (V2) is 600 m from the mine, and village 3 (V3) is approximately 1 km from the mine. There are 32, 38 and 30 households in V1, V2 and V3 respectively, and they have similar population structures, living conditions and lifestyle. The geographical locations of the three sampled villages are shown in Figure 1.

### Sampling

The drinking water of the 3 villages is tap water, which is provided by the same water supply company. We collected 10 samples from local families at random to represent the whole area. The water samples were collected in precleaned polyethylene bottles. After acidification with HCl, the samples were placed in an ice bath, then transported to the laboratory and kept at −20°C until they were analyzed.

5 soil samples were collected at the edges of residential streets and crop fields in each village. All soil samples were taken from the upper 5 cm of ground and stored in polyethylene bags. Each sample (1 kg approximately) consisted of 5 subsamples collected in an area of 100 m^2^, pooled and homogenized to form a representative sample. The samples were air-dried, and stones and coarse plant roots or residues were removed. They were then thoroughly mixed, crushed, passed through a 2-mm mesh sieve and a 0.149-mm mesh sieve, and then stored in polyethylene bottles at ambient temperature prior to chemical analysis.

From January to March 2011, air sampling was conducted simultaneously in the 3 villages. 20 samples in each village and total 60 indoor and outdoor samples were collected at different sites in and around the study area. The indoor air samples were obtained from the rooms of randomly chosen families. The air samples were collected onto filter membranes using a PM_10_ air sampler (Tianhong instruments Co. Ltd, Wuhan, China) run for 3 h at a flow rate of 100 L/min. For the detection of trace Hg, the sulfhydryl cotton enrichment method was used.

In each village, we selected 4 fields at different directions, and locally produced vegetable samples were harvested in double in a quadrant of 0.25 m^2^ randomly selected from each site. In total, 24 vegetable samples were collected in the study area. Food produced out of town, including rice, pork and eggs, was collected from the markets. The samples were cut into small pieces after thoroughly washing with tap water and deionized water. After drying to constant weight at 80°C in an oven, the food samples were ground and sieved for acid digestion.

### Human Exposure Survey

A questionnaire-based survey was conducted in the studied villages to determine key risk factors such as dietary behaviors, daily activities and lifestyle of local people. We invited 50 local residents to participate the survey in each village. All participants were selected randomly. In V1, the research staff obtained written informed consent from 42 participants who are permanent residents of this village, and there were 42 participants in V2 and 36 participants in V3. The environmental samples were collected from these participants’ immediate surroundings.

To characterize the exposure level and metal accumulation in human body, hair samples were also taken from the above survey participants. Twenty-nine, 30 and 13 participants provided usable samples in V1, V2 and V3, respectively. The hair samples were analyzed for the 10 metals to characterize the in vivo exposure levels and potential differences among the different indigenous groups in the study area.

### Analytical Methods

Except for Hg, the USEPA 200.8 method was used to determine the trace metals in the water and soil samples, which were measured with inductively coupled plasma-mass spectrometry (ICP-MS) (Agilent 7500i, Agilent Scientific Technology Ltd., USA). For the airborne particle samples, an atomic absorption spectrometer (AAS, Vario 6, Jena Co., Ltd., Germany) was used to analyze the filters loaded with heavy metals according to the standard examination method for ambient air in China. Using the USEPA 6020A method, the concentrations of metals in the food and hair samples were determined with ICP-MS. The determination of Hg in the water, soil, food and hair samples was conducted using thermal decomposition, amalgamation and atomic absorption spectrophotometry (TDA/AAS) (AMA 254, LECO Co., Ltd., USA) according to the USEPA 7473 method. Trace Hg in the air, enriched in sulfhydryl cotton, was determined using cold vapor atomic absorption spectrophotometry (CVAAS) (JKG-205, Jilin Scientific Technology Co., Ltd., China) according to the NIOSH 6009 method. The detection limit of the measurements was defined as the concentration value, which is numerically equal to three times the standard deviation of 10 replicate blank measurements. Reagent blanks and standard reference materials were used in the analysis for quality assurance and quality control. The recoveries of the elements ranged from 90% to 110%.

### Risk Calculation

The average daily intake (ADI) of metals by the human subjects was calculated using the following equation, which is recommended by the USEPA [37]. The equation links the time-averaged dose to the exposure medium concentration.
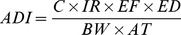
(1)where ADI is the average daily intake or dose through ingestion or inhalation (mg/kg-day); C is the chemical concentration in the exposure medium (mg/L, mg/kg, or mg/m^3^); IR is the ingestion rate (L/day, kg/day, or m^3^/day); EF is the exposure frequency (day/year); ED is the exposure duration (year); BW is the body weight (kg) and AT is the time period over which the dose is averaged (day).



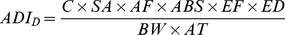
(2)For exposure dose through dermal contact (ADI_D_) calculation, *SA* is the exposed skin surface area (cm^2^), *AF* is the adherence factor (mg/cm^2^/day), and *ABS* is the dermal absorption factor.

To calculate the exposure dose of each pathway, the ingestion rate of drinking water and food were obtained through the above questionnaire-based exposure survey. In addition, the ingestion rate of soil, inhalation rate of air, the adherence factor (AF), and the dermal absorption factor (ABS) were obtained from open database and literature [38], [39].

The human health risks posed by heavy metal exposure are usually characterized by the hazard quotient (HQ) [37], the ratio of average daily intake to the reference dose (RfD) or the reference concentration in air (RfC) for an individual pathway and chemical. A quotient under 1 is assumed to be safe. When the HQ value exceeds 1, there may be concerns for potential health risks associated with overexposure. The RfD and RfC values used in this study were recommended by open chemical databases and shown in Table 1. To assess the overall potential for health effects posed by more than one metal, summing HQs across metals can serve as a conservative assessment tool to estimate high-end risk rather than low-end risk to protect the public. In this study, the total HQ was used as a screening value to identify whether there is significant risk caused by metals, and any difference of total health risk existed among the 3 villages.

**Table 1:** Metal reference doses (RfD).

Metal	RfD (mg/kg-d)	Source	RfC (mg/m^3^)	Source
Ag	5.0E−3	IRIS^a^	−	
Cd	1.0E−3	IRIS	1.0E−5	ATSDR^b^
Cr	1.5E + 0	IRIS	−	
Cu	4.0E−2	HEAST^c^	−	
Ni	2.0E−2	IRIS	9.0E−5	ATSDR
Pb	1.4E−4	Oak Ridge^d^	−	
Se	5.0E−3	IRIS	2.0E−2	Cal EPA^e^
Tl	3.0E−6	IRIS	−	
Zn	3.0E−1	IRIS	−	
Hg	1.6E−4	Cal EPA	3.0E−4	IRIS

a Integrated Risk Information System, U.S. EPA;

b The Agency for Toxic Substances and Disease Registry, U.S.;

c Health Effects Assessment Summary Tables, U.S. EPA;

d Oak Ridge National Laboratory, U.S.;

e California Environmental Protection Agency, U.S.

To accommodate the uncertainties associated within the calculation process, Monte Carlo simulation technique was used based on Crystalball software (Oracle Corporation, Vallejo, US) and considering 10,000 iterations. Before this process, distribution characteristics of each exposure parameter were tested according to the exposure survey results. A probabilistic distribution of the exposure dose and HQ values was then obtained as simulation result.

## Results

### Heavy Metal Levels in Different Environmental Media

The analysis results show that the tap water in the studied area is safe (unpublished data, Table S1), as all the trace heavy metal concentrations are far less than the national drinking water quality criteria.

Indoor air samples were obtained from 13, 15, and 12 households in V1, V2, and V3, respectively. The chemical analysis results (unpublished data, Table S2) show that Cu, Zn and Pb are the main pollutants. V1 was the most seriously polluted among the studied villages. The average metal levels there were 6.1 µg/m^3^ for Cu, 2.5 µg/m^3^ for Zn and 2.1 µg/m^3^ for Pb, with a detection rate of nearly 100%. The chemical analysis results for the outdoor air show that Zn and Pb have the highest concentrations. In V1, the median metal levels were 4.2 µg/m^3^ for Zn and 2.4 µg/m^3^ for Pb, with a detection rate of 93.8% and 100% respectively.

The chemical analysis results of the soil samples show that 10 PP metals were detected in all samples (unpublished data, Table S3). In general, V1 is the most seriously polluted, followed by V2, and the soil quality is good in V3. In V1, the soil Pb concentration is as high as 2507 mg/kg, and the average soil zinc concentration as high as 9281 mg/kg, which is 100 times greater than that of V3.

It is well known that vegetables absorb metals from the soil. The self-produced vegetables contained higher metal concentrations in V1, especially of Zn and Pb. The average Zn concentrations in pakchoi in V1, V2 and V3 were 10.48, 5.24 and 2.34 mg/kg, respectively. The average Pb concentrations reached 0.24 and 0.08 mg/kg in V1 and V2, respectively, whereas Pb was not detected in V3. Different from the vegetables, the metals could not be detected in most of the imported egg, pork and rice samples.

### Exposure Factors

The demographic, activity and lifestyle questionnaire results are listed in Table 2. The periods that local residents have lived in this mining area range from 2 to 35 years. Local residents spend more time indoors than outdoors. The people in this rice-producing region mainly feed on rice, pakchoi, cabbage and pork. The local people almost do not eat flour, whereas the national average level was 140 g/person/day [40]. However, the consumption rate of rice for the local people was 360 g/person/day, much greater than the national average level as 238 g/person/day [40]. The consumption rate of vegetables, pakchoi dominantly, for the local people was 310 g/person/day, higher than the national average level as 276 g/person/day [40]. In this area, the vegetables are mostly self-grown, whereas other foodstuffs are mainly purchased from the market. Additionally, no difference of demographic, activity and lifestyle characteristics among these 3 villages was found at such spatial scale.

**Table 2:** Demographic, lifestyle, and dietary characteristics of the local residents.

Characteristics	Mean	SD	Unit
Weight	62.58	10.23	kg
Activity			
Indoors	8.47	3.99	h
Outdoors	5.84	3.98	h
Sleep	9.24	2.14	h
Dietary			
Rice	360.29	71.92	g/d
Flour	6.29	17.38	g/d
Pakchoi	171.76	82.74	g/d
Cabbage	63.53	38.66	g/d
Spinach	30.44	19.36	g/d
Celery	24.26	11.49	g/d
Pork	56.85	25.01	g/d
Egg	45.44	25.97	g/d
Water ingestion	1.88	0.62	L/d
Exposure duration	35.35	18.68	a

SD: Standard deviation.

### Health Risks

**Figure 2:**
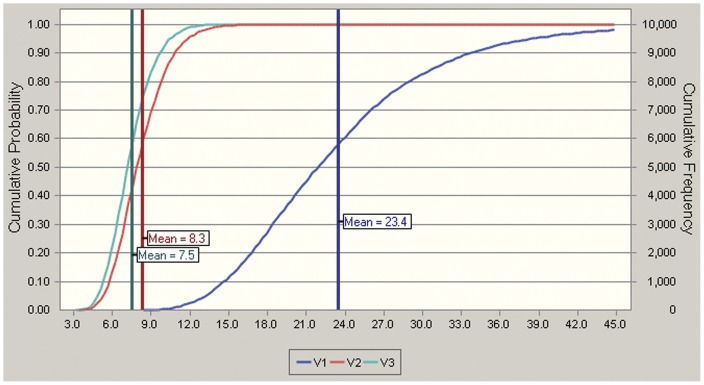
Cumulative probability distribution of the total HQs in the three villages. The health risks were evaluated by means of a Monte Carlo simulation based on Crystalball software for 10,000 iterations.

The health risks for the local population due to exposure to metals were evaluated using Monte Carlo simulation. The cumulative probability distribution of the total HQ values in the three villages is presented in Figure 2. For V1, the HQ ranged from 6.8 to 81.3; for V2, the HQ ranged from 2.9 to 19.3; and for V3, the HQ ranged from 3.3 to 15.8. The mean HQ values of V1, V2 and V3 were 23.4, 8.3 and 7.5, respectively. Obviously, the HQ of V1 was much higher than those of the other two.

The heavy metal HQ values from dietary sources, dust inhalation (both indoor and outdoor air), water ingestion and dermal contact, and soil ingestion and dermal contact were aggregated. The Monte Carlo simulated health risk assessment results are shown in Table 3. There is a significant difference of Pb, Cd and Hg HQs among the three villages. In V1, the average HQ value of Pb is as high as 16.2, indicating that the chronic daily intake dose exceeds the safe reference dose by 15 fold. The HQ values of Pb in V2 and V3 reach 3.6 and 3.0, respectively. The chronic daily intake dose of Cd and Hg also exceeds the corresponding limits, and the HQ in V1 was greater than those in V2 and V3.

**Table 3:** Hazard quotients of different metals in each village.

Metal	V1		V2		V3	
	Mean	SD	Mean	SD	Mean	SD
Ag	0.0009	0.0005	0.0003	0.0001	0.0002	0
Cd	3.32	1.91	1.49	0.843	1.81	0.812
Cr	0.001	0.0008	0.001	0.0005	0.0009	0.0005
Cu	0.189	0.046	0.164	0.039	0.18	0.046
Ni	0.42	0.15	0.41	0.15	0.35	0.15
Pb	16.2	6.96	3.63	1.33	3.01	0.92
Se	0.018	0.015	0.018	0.016	0.019	0.016
Tl	0.17	0.062	0.058	0.023	0.097	0.13
Zn	0.30	0.076	0.29	0.073	0.28	0.073
Hg	2.84	1.06	2.28	0.65	1.83	0.50

**Figure 3:**
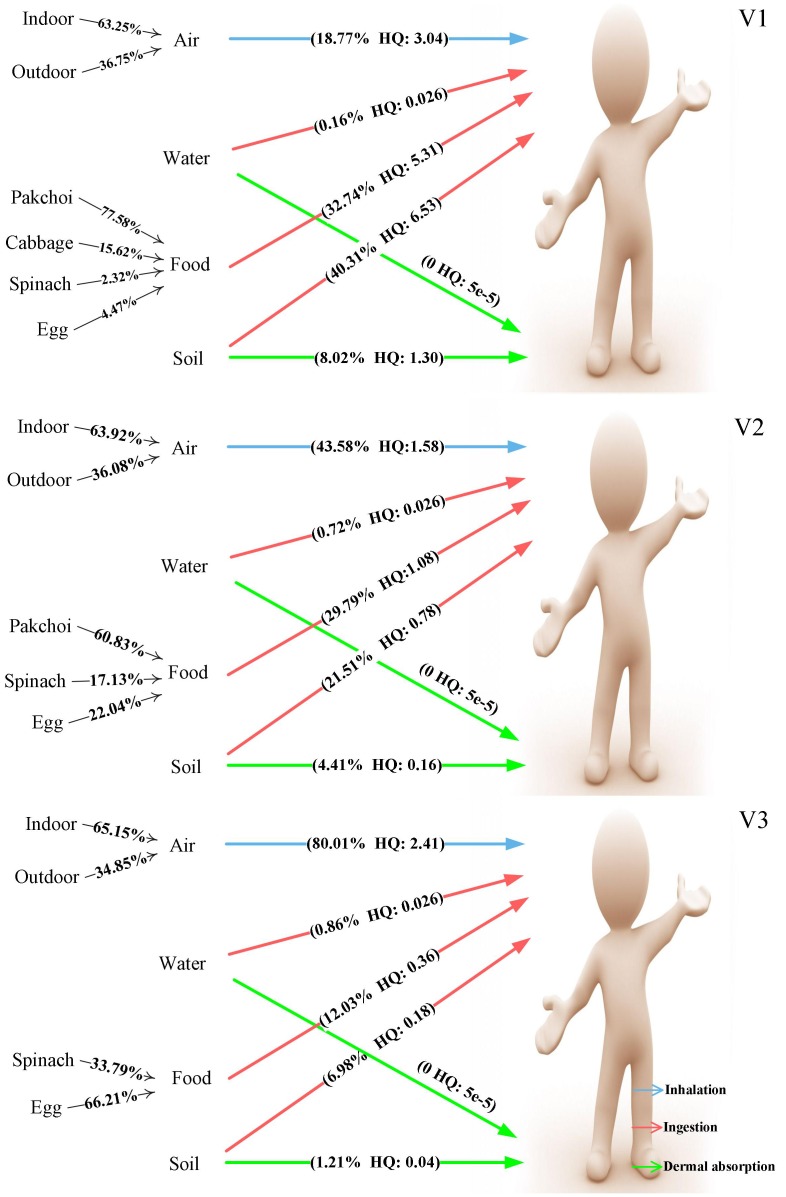
Multipathway analysis of HQ (Pb). Each pathway’s contribution to total Pb exposure of local residents in the three studied villages was calculated based on average HQ values.

As Figure 3 shows, soil ingestion was the primary pathway to Pb exposure for V1, accounting for 40.3% of the total HQ. Food consumption contributed nearly one-third to the total HQ, and among different food sources, pakchoi were the most significant, contributing 77%. Inhalation contributed nearly one-fifth to the total HQ. Furthermore, indoor air contributed twice as much as did outdoor air. For the other two villages, the proportion of inhalation increased, whereas those of food ingestion and soil ingestion decreased sharply. This result is consistent with trend of Pb levels in the soil with increasing distance from the mine.

**Figure 4:**
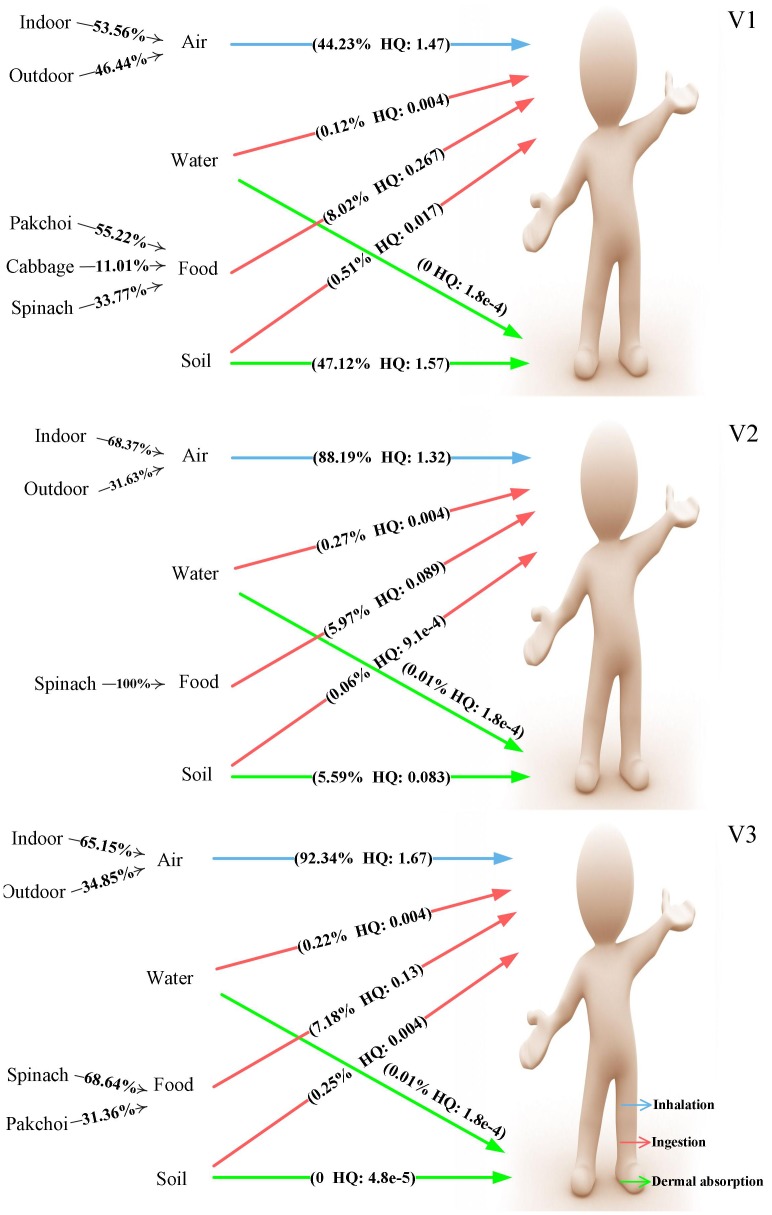
Multipathway analysis of HQ (Cd). Each pathway’s contribution to total Cd exposure of local residents in the three studied villages was calculated based on average HQ values.

For Cd (Figure 4), the exposure to and uptake of this non-essential element was mainly from soil dermal contact, which contributed 47.1% to the total HQ in V1. Inhalation also contributed 44.3% to the total risk, and indoor air contributed slightly more than outdoor air. However, in V2 and V3, the total HQ decreased with increasing distance from the mine, and the contribution of soil dermal contact decreased sharply with an evident increase of inhalation. The ingestion of water and food is safe for local residents.

**Figure 5:**
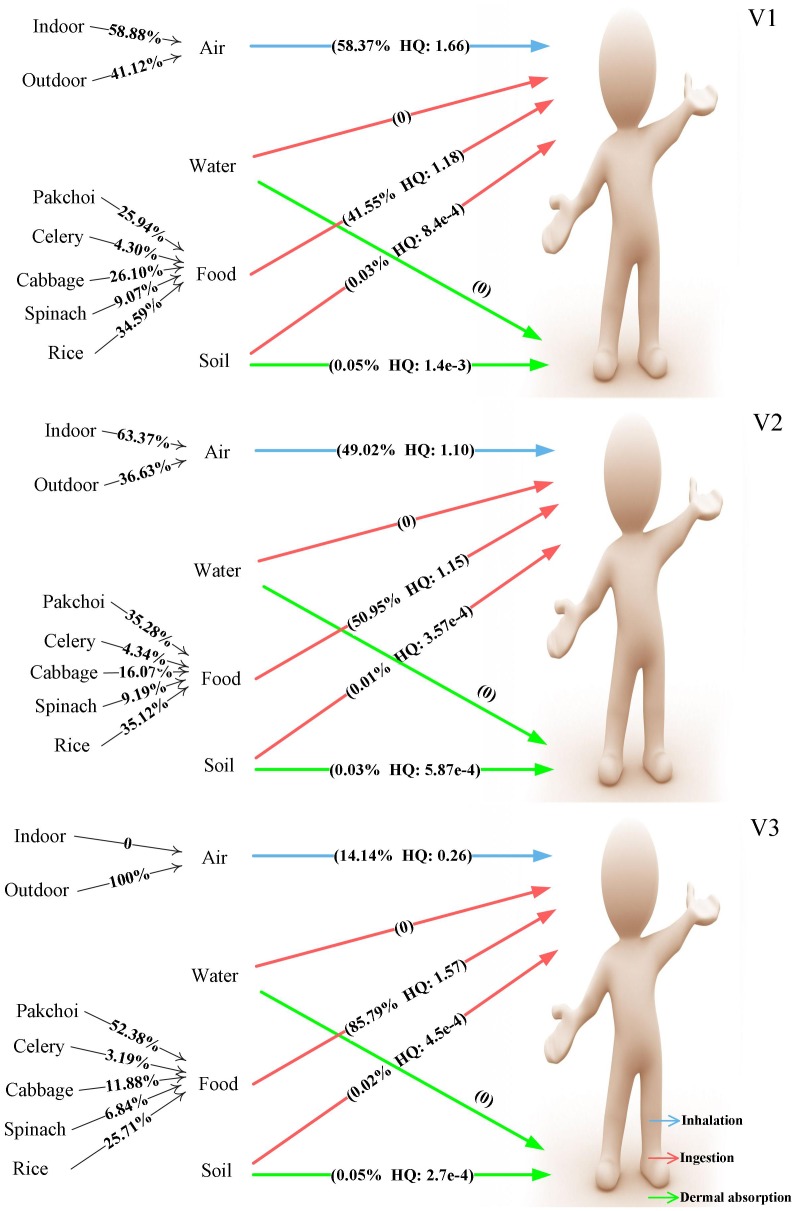
Multipathway analysis of HQ (Hg). Each pathway’s contribution to total Hg exposure of local residents in the three studied villages was calculated based on average HQ values.

The local residents’ average daily exposure to Hg also exceeded the safe reference dose, and the HQ value of the residents in V1 was the highest. Taking V1 as an example, the multipathway analysis (Figure 5) showed that the people of that village are exposed to Hg mainly through inhalation, with indoor air contributing the most. Food also contributed 41.6% to the total risk, mainly due to self-produced broad-leaf vegetables, such as pakchoi and cabbage, whereas the risk posed by rice from the market was not trivial. Different from Pb and Cd, soil pollution only contributes a minor fraction. V2 and V3 had similar profiles of metal levels.

### Heavy Metal Levels in Hair

The metal concentrations in the hair samples are shown in Table 4. Nine metals were detected in the local residents’ hair samples. The concentration of Zn was the highest of the detected metals. The geometric mean of Zn reached 240 mg/kg, which is much greater than those of the other metals. The hair samples from the residents in V1 contained higher Pb (8.14 mg/kg) and Hg (0.86 mg/kg) concentrations than those from V2 and V3 (P<0.05). This result is consistent with the variation of the HQ values. For the other metals, no significant differences or strong relationships with the HQ were found among the three villages.

**Table 4:** Heavy metal levels in hair (mg/kg).

Metals	LOD	V1		V2		V3	
		Mean(SD)	Detected proportion	Mean (SD)	Detected proportion	Mean (SD)	Detected proportion
Ag	0.05	-	0	-	0	-	0
Cd	0.01	0.15(0.42)	90.0%	0.08(0.12)	63. 3%	0.14(0.17)	91.7%
Cr	0.05	1.60(1.05)	100%	2.68(2.47)	100%	2.46(5.20)	100%
Cu	0.05	9.12(7.88)	100%	11.13(13.38)	100%	8.86(3.52)	100%
Ni	0.05	0.44(0.75)	86.7%	0.73(0.89)	100%	0.71(0.90)	91.7%
Pb	0.05	8.14(12.30)	100%	2.26(4.19)	100%	3.98(4.69)	100%
Se	0.25	0.58(0.37)	46.7%	0.77(0.94)	76.7%	0.81(0.86)	75.0%
Tl	0.05	0.07(0.01)	6.7%	0.09(0.01)	6.7%	0.10(0.05)	41.7%
Zn	0.25	270.3(441.1)	100%	241(262)	100%	201.4(126.1)	100%
Hg	0.01	0.86(5.00)	100%	0.59(1.55)	100%	0.55(1.03)	75.0%

LOD: Limit of detection.

## Discussion

The environmental sample analysis results showed widespread heavy metal contamination in the air, especially of Pb, Zn and Cu. The nearest village (V1) to the mine had the heaviest pollution. Due to its respiratory, neurological, and potential carcinogenic effects, Pb is regulated by the National Ambient Air Quality Standards with seasonal mean levels not to exceed 1.0 µg/m^3^ and annual mean levels not to exceed 0.5 µg/m^3^ [41]. The proposed WHO guideline for Pb is 0.5 µg/m^3^ [42]. The average Pb concentrations in the three villages all exceeded the limit. The highest Pb concentration occurred in the indoor air in V1, which reached 5.9 µg/m^3^, and this concentration was approximately 6-fold that of the national limit.

Although there was no significant difference between indoor and outdoor air in general, the highest metal concentration was detected in indoor air. This might be attributed to various indoor heavy metal pollution emissions. Some indoor particles come from the infiltration of outdoor air or are derived from material inside the house such as household plant material, paint chips, flakes of construction materials, fuel burning and smoking [43], [44], [45]. A previous study found that resuspension has a significant impact on indoor particle concentrations. For example, simply walking into a room can increase the particle concentration by 100% for some supermicron particle sizes [43].

Distance from the mine was a key determinant of soil pollution, as there are significant spatial differences among these three villages. This finding was consistent with the conclusions of previous studies [46]. There was heavy Pb, Zn, Cu and Cd contamination in V1, the nearest village to the mine and the most polluted. The main body of this large mine is underground, whereas the sharp decline of heavy metal concentrations with increasing distance to the mining area suggests that it is the dominant source of metals such as Pb and Zn in the soil. This result was consistent with the previous studies, revealing the fly ash emitted from the ventilation outlet of the mine factory was the pollution source in this area [47]. Additionally, the run-off loss of solid ore deposited on open ground and dust emission from the transportation are likely important pollution sources too.

In addition, the relatively higher Pb and Zn concentrations in the self-produced vegetables were consistent with the concentrations in the top soil. In general, the highest metal concentrations were found in green leafy vegetables [18]. The deposition from dust onto the foliage of these vegetables would also add to the heavy metal content because metals firmly attach to the surfaces [48]. In this study, self-produced pakchoi and Chinese cabbage, especially in V1, had much higher Pb concentrations than did the other vegetables. In contrast, eggs, pork and rice bought from market were safer, as they had much lower metal concentrations than those of the vegetables. It was evident that outside food was safer than the self-produced food.

The calculated HQ values for all three villages exceeded the limits, indicating heavy metal pollution may pose high potential health risk to the people within the vicinity of the lead-zinc mining area. The factory itself declared to comply with strict environmental protection requirements with the goal of zero pollutants emission. The local environmental protection agency had set up hygienic buffer zone with a 200 meters safety protection distance around the mine. However, this regulation was not strictly followed, for the mine factory was only 100 meters from V1. The HQ in V1, which reached 23.4, was significantly higher than those of the other two villages. The average HQs of V2 and V3 also reached 8.3 and 7.5, respectively, indicating that the 200-m safety protection distance failed to protect the public’s health. Furthermore, the estimated risks were mainly due to Pb, Cd and Hg. In V1, the average HQ value of Pb was as high as 16.2, accounting for 65% of the total HQ. Pb also contributed nearly half of the average HQ in the other two villages. The chronic daily intake dose of Cd and Hg also exceeded the safe level. Despite Zn having the highest concentration among the metals, its HQ value was below the limit, indicating that Zn does not pose a health risk to the local residents, as Zn is an essential element for the human body, and only superfluous ingestion can cause adverse health effects. It’s worth noting that, different from soil exposure, which declined sharply when the distance to the mine increased, as to the pathway of inhalation, V3 had higher Pb and Cd HQ when compared to V2 in average. However, there were no significant differences (P>0.05). More environment examinations maybe needed in the future to assess health risk posed by Pb and Cd air pollution.

With regard to each metal, different exposure pathways made different contributions in different locations. Pb posed the highest estimated risk, which was mainly from soil ingestion, indoor air inhalation and pakchoi ingestion. However, in V1, the general population was primarily exposed to Cd via soil dermal contact and inhalation. As to Hg, food ingestion and inhalation contributed most to the HQ. In this study, the average indoor and outdoor air concentrations in V1 reached 734 and 543 ng/m^3^ respectively, and were much higher than the national background level, 6 ng/m^3^ [49]. Accordingly, Hg intake through inhalation is the main and most important Hg exposure source. This finding was in accord with a previous study in Guizhou Province, where atmospheric Hg concentration around power plants ranged from 556.7 to more than 1000 ng/m^3^, and the local residents have the potential of developing adverse health problems because of over exposure [50]. Additionally, Hg content in rice in the study area was 0.03 mg/kg, which was lower than that of Guizhou in China (0.48 mg/kg), but much higher than that in Taiwan (0.001 mg/kg) and Shanghai (0.0047–0.0056 mg/kg) [51], [52], [53]. The mean concentration of vegetables measured in the study area range from 0.02 to 0.05 mg/kg, which was a bit lower than those measured at Nanning (0.024–0.077 mg/kg), but much higher than those measured at Shanghai (0.0005–0.006 mg/kg) and Zhongshan (0.0023–0.010 mg/kg) in China [54], [55], [56]. In general, the study area had higher Hg concentrations in rice and vegetables among the areas compared, verifying that intake of rice and vegetables is another important Hg exposure source for the residents in the study area.

Generally, the inhalation of indoor air usually contributed more than that of outdoor air because of the relatively heavier indoor air pollution and the amount of time local people spent on indoor activities. The contributions of dermal contact and ingestion of soil declined sharply with increasing distance from the mine, especially for Pb and Cd. This result was in accordance with the spatial trend of metal levels in the soil. Self-grown pakchoi was an important risk source for local residents, specifically in V1, due to its relatively high metal concentrations and large proportion in the dietary structure. These results show that dermal contact, the ingestion of soil, the inhalation of indoor air and vegetable consumption are important in reducing the overall health risk. Human exposure to soil, air and self-produced vegetables indicates the necessity of future policy responses and interventions. Local residents, especially in V1, should reduce their consumption of self-grown vegetables, maintain clean indoor air, and avoid contact with the polluted soil and dust.

Except for Ag, 9 PP metals were detected in the human hair samples. Among these detected metals, the Zn content in the hair of the sampled individuals was found to be the highest and exceeded 200 mg/kg. However, no differences were found among various locations. Consistent with the higher HQ value of the residents in V1, their hair samples exhibited higher, statistically significant Pb (8.14 mg/kg) and Hg (0.86 mg/kg) levels (P<0.05) than did the other two villages. The average Pb concentration in V1 was lower than that of an electronic waste recycling area in Taizhou (49.5 mg/kg), Jiangsu Province, China, but higher than that of the control sites in Ningbo (2.53 mg/kg) and Shaoxing (6.61 mg/kg) in a previous study [31]. The highest Pb concentration in hair found in V1, V2 and V3 reached 48.0, 19.7 and 18.0 mg/kg respectively, which exceeded the suggested upper limit of normal value of hair Pb (10.0 mg/kg) [57]. The HQ and corresponding Pb and Hg concentrations in the hair declined with increasing distance from the mine. It can be stated that the heavy metal pollution from the lead-zinc mine increased the exposure level of local residents, especially in V1. Human hair could be a useful biomonitoring tool to assess the extent of Pb and Hg exposure to residents in metal-polluted areas. However, for Zn, Cu, and Cd, higher levels in the hair were not detected in V1, where higher estimated exposure levels were found. This showed that hair was not biomonitoring indicator of exposure for all elements. More studies should be done to better justify use of hair as biomonitoring indicator for other metals considered in this area.

According to the above analysis, it can be concluded that mining activity may pose high potential risk to the public, and current safety protection distance failed to protect the public’s health. Most of the estimated risks came from soil, self-produced vegetables and indoor air inhalation. In summary, our results suggest that the exposure of local inhabitants to heavy metals in the mining area is through multipathways. This study highlights the importance of site-specific multipathway risk assessment. However, it should also be pointed out that our risk model used conservative assumptions and we were not able to conduct a human health impacts analysis. More detailed and in-depth health investigation is necessary in the future to examine whether adverse health outcomes occur, and provide decision-making support for pollution control in this metal-polluted area accordingly.

## Supporting Information

Table S1 Metals in tap water (µg/L).
(DOC)

Table S2 Metal concentration in air samples (mg/m^3^).
(DOC)

Table S3 Metals in soil samples (mg/kg).
(DOC)

Table S4 Metals in vegetable samples (mg/kg, fresh weight).
(DOC)
