# Supplementary material for: Human Exposure Pathways of Heavy Metals in a Lead-Zinc Mining Area, Jiangsu Province, China
Source: PLoS One. 2012 Nov 13;7(11):e46793. doi: 10.1371/journal.pone.0046793 (PMC3496726; doi:10.1371/journal.pone.0046793)
Supplement: Table S1 — Metals in tap water (µg/L). (DOC) [file pone.0046793.s001.doc]

Table S1 Metals in tap water (μg/L)

| **Metal** | **LOD** | **Range** | **Mean** | **SD** | **National limit** |
| --- | --- | --- | --- | --- | --- |
| Ag | 1.0 | ND | - | - | - |
| Cd | 0.1 | 0.1−0.2 | 0.2 | 0.1 | 5 |
| Cr | 1.0 | ND | - | - | - |
| Cu | 0.2 | 1.4−3.2 | 1.9 | 0.6 | 1000 |
| Ni | 0.1 | 0.8−1.0 | 0.9 | 0.1 | 20 |
| Pb | 0.1 | 0.1−0.2 | 0.1 | 0.1 | 10 |
| Se | 5.0 | ND | - | - | 10 |
| Tl | 0.1 | ND | - | - | 0.1 |
| Zn | 1.0 | 10.0−266.0 | 128.6 | 102.6 | 1000 |
| Hg | 0.1 | ND | - | - | 1 |

LOD: limit of detection; ND: not detected; SD: standard deviation.
