# Supplementary material for: Human Exposure Pathways of Heavy Metals in a Lead-Zinc Mining Area, Jiangsu Province, China
Source: PLoS One. 2012 Nov 13;7(11):e46793. doi: 10.1371/journal.pone.0046793 (PMC3496726; doi:10.1371/journal.pone.0046793)
Supplement: Table S2 — Metal concentration in air samples (mg/m3). (DOC) [file pone.0046793.s002.doc]

Table S2 Metal concentration in air samples (mg/m3)

| **Metal** | **LOD** | **V1** | | | | **V2** | | | | **V3** | | | |
| --- | --- | --- | --- | --- | --- | --- | --- | --- | --- | --- | --- | --- | --- |
|  |  | **indoor** | | **outdoor** | | **indoor** | | **outdoor** | | **indoor** | | **outdoor** | |
|  |  | Range | Mean± SD | Range | Mean± SD | Range | Mean± SD | Range | Mean± SD | Range | Mean± SD | Range | Mean± SD |
| Ag | 6.0e-6 | ND−1.2e-5 | 7.1e-6±3.5e-6 | ND−8.9e-6 | 7.9e-6±2.6e-6 | ND−1.2e-5 | 8.7e-6±2.1e-6 | ND | − | ND−1.0e-5 | 7.9e-6±1.3e-6 | ND | − |
| Cd | 3.0e-6 | ND−4.7e-5 | 2.7e-5±1.7e-5 | ND−3.0e-5 | 2.5e-5±7.8e-6 | ND−6.3e-5 | 2.5e-5±2.4e-5 | ND−3.4e-5 | 1.5e-5±1.6e-5 | ND−4.9e-5 | 2.7e-5±1.2e-5 | ND−4.4e-5 | 2.0e-5±1.7e-5 |
| Cr | 4.0e-5 | ND−1.5e-4 | 8.3e-5±3.4e-5 | 4.7e-5−8.8e-5 | 6.1e-5±1.8e-5 | ND−8.0e-5 | 4.5e-5±2.1e-5 | 4.1e-5−9.9e-5 | 6.2e-5± 2.5e-5 | ND−1.5e-4 | 5.2e-5±3.5e-5 | ND−1.5e-4 | 6.6e-5±4.8e-5 |
| Cu | 8.0e-5 | 1.0e-3−1.5e-2 | 6.1e-3±5.7e-3 | ND−2.8e-4 | 2.1e-4±5.8e-5 | 1.9e-4−1.8e-2 | 5.1e-3±5.3e-3 | ND−1.6e-4 | 1.1e-4±5.2e-5 | 5.5e-4−2.0e-2 | 7.2e-3±7.2e-3 | ND−5.4e-4 | 1.9e-4±1.9e-4 |
| Ni | 2.0e-5 | ND−6.7e-5 | 4.3e-5±2.7e-5 | ND−3.9e-5 | 2.7e-5±1.1e-5 | ND−6.7e-5 | 3.6e-5±2.2e-5 | ND−6.0e-5 | 5.3e-5±9.2e-6 | ND−7.8e-5 | 1.7e-5±1.8e-5 | ND−7.3e-5 | 2.1e-5±1.0e-5 |
| Pb | 2.0e-4 | 2.2e-4−5.9e-3 | 2.1e-3±1.8e-3 | 6.7e-4−4.1e-3 | 2.4e-3±1.5e-3 | 2.1e-4−2.8e-3 | 1.1e-3±8.1e-4 | 2.7e-4−2.8e-3 | 1.3e-3±1.2e-3 | 5.8e-4−2.3e-3 | 1.7e-3±5.8e-4 | ND−2.8e-3 | 1.7e-3±9.8e-4 |
| Se | 1.5e-5 | ND−2.7e-5 | 2.1e-5±8.5e-6 | ND−2.1e-5 | 1.7e-5±4.9e-6 | ND−3.5e-5 | 2.2e-5±7.7e-6 | ND−4.3e-5 | 3.5e-5±1.1e-5 | ND−4.3e-5 | 3.1e-5±8.6e-6 | ND−4.6e-5 | 3.3e-5±1.1e-5 |
| Tl | 3.0e-6 | ND−3.1e-6 | 1.6e-6±3.8e-7 | ND−3.4e-6 | 2.0e-6±8.2e-7 | ND−1.6e-5 | 9.5e-6±3.7e-6 | ND−1.5e-5 | 4.9e-6±5.8e-6 | ND−9.8e-4 | 1.0e-4±2.9e-4 | ND−2.3e-5 | 1.6e-5±6.1e-6 |
| Zn | 2.0e-4 | 2.7e-4−7.0e-3 | 2.5e-3±4.1e-3 | 1.4e-3−6.5e-3 | 4.2e-3±2.8e-3 | 2.1e-4−1.7e-3 | 6.5e-4±4.2e-4 | 2.0e-4−1.8e-3 | 9.3e-4±8.1e-4 | 2.0e-4−1.3e-3 | 1.1e-3±9.8e-4 | 2.9e-4−2.1e-3 | 9.9e-4±6.3e-4 |
| Hg | 1.7e-4 | ND−6.6e-3 | 7.3e-4±5.3e-4 | ND−7.5e-4 | 5.4e-4±2.8e-4 | ND−6.8e-4 | 5.9e-4±7.1e-5 | ND−1.1e-3 | 7.8e-4±3.8e-4 | ND | − | ND−6.4e-4 | 2.8e-4±2.6e-4 |
