# Supplementary material for: Human Exposure Pathways of Heavy Metals in a Lead-Zinc Mining Area, Jiangsu Province, China
Source: PLoS One. 2012 Nov 13;7(11):e46793. doi: 10.1371/journal.pone.0046793 (PMC3496726; doi:10.1371/journal.pone.0046793)
Supplement: Table S3 — Metals in soil samples (mg/kg). (DOC) [file pone.0046793.s003.doc]

Table S3 Metals in soil samples (mg/kg)

| **Metal** | **LOD** | **V1** | | | **V2** | | | **V3** | | |
| --- | --- | --- | --- | --- | --- | --- | --- | --- | --- | --- |
|  |  | **Range** | **Mean** | **SD** | **Range** | **Mean** | **SD** | **Range** | **Mean** | **SD** |
| Ag | 0.1 | 2.7−22.6 | 9.8 | 8.7 | 0.4−2.6 | 1.1 | 0.9 | ND | - | - |
| Cd | 0.01 | 4.3−129.0 | 49.7 | 57.4 | 0.5−8.9 | 2.6 | 3.6 | 7.0e-2−1.5e-1 | 0.1 | 3.0e-2 |
| Cr | 0.1 | 16.8−65.4 | 33.6 | 20.3 | 2.5−26.4 | 23.4 | 2.5 | 18.7−23.7 | 21.4 | 2.2 |
| Cu | 0.1 | 46.8−611.0 | 261.7 | 236.6 | 23.3−86.3 | 47.7 | 23.2 | 21.9−29.4 | 25.5 | 2.6 |
| Ni | 0.1 | 15.1−27.0 | 21.9 | 4.7 | 1.6−24.7 | 22.9 | 1.6 | 17.2−21.9 | 19.9 | 2.7 |
| Pb | 0.1 | 4.4e2−6.1e3 | 2.5e3 | 2.3e3 | 9.4e1−8.9e2 | 300.2 | 3.3e2 | 43.9−73.9 | 62.8 | 11.9 |
| Se | 0.5 | 0.7−3.1 | 1.7 | 1.0 | 0.2−1.0 | 0.7 | 0.23 | ND | - | - |
| Tl | 0.1 | 0.14−0.9 | 0.7 | 0.1 | 8.0e-2−4e-1 | 0.4 | 0.08 | 0.1−0.2 | 0.1 | 1.0e-2 |
| Zn | 0.5 | 8.6e2−2.4e4 | 9.3e3 | 1.1e4 | 1.6e2−2.0e3 | 6.5e2 | 7.7e2 | 69.3−86.7 | 79.2 | 6.6 |
| Hg | 0.01 | 0.1−0.7 | 0.4 | 0.24 | 4.0e-2−1.7e-1 | 0.2 | 4.04-2 | 5.0e-2−1.5e-1 | 0.1 | 4.0e-2 |
