# Supplementary material for: Human Exposure Pathways of Heavy Metals in a Lead-Zinc Mining Area, Jiangsu Province, China
Source: PLoS One. 2012 Nov 13;7(11):e46793. doi: 10.1371/journal.pone.0046793 (PMC3496726; doi:10.1371/journal.pone.0046793)
Supplement: Table S4 — Metals in vegetable samples (mg/kg, fresh weight). (DOC) [file pone.0046793.s004.doc]

Table S4 Metals in vegetable samples (mg/kg, fresh weight)

| **Metal** | **LOD** | **V1** | | | **V2** | | | **V3** | | |
| --- | --- | --- | --- | --- | --- | --- | --- | --- | --- | --- |
|  |  | **Pakchoi** | **Cabbage** | **Spinach** | **Pakchoi** | **Cabbage** | **Spinach** | **Pakchoi** | **Cabbage** | **Spinach** |
| Ag | 0.05 | ND | ND | ND | ND | ND | ND | ND | ND | ND |
| Cd | 0.01 | 0.03-0.08 | ND-0.07 | ND-0.16 | ND | ND | ND-0.14 | ND-0.04 | ND | ND-0.31 |
| Cr | 0.05 | ND-0.24 | ND-0.39 | ND-0.09 | ND-0.21 | ND-0.36 | ND-0.09 | ND-0.24 | ND-0.12 | ND-0.08 |
| Cu | 0.05 | 0.10-0.50 | ND-0.19 | ND-1.71 | 0.17-0.29 | ND-0.09 | ND-1.24 | 0.17-0.19 | ND-0.17 | ND-1.43 |
| Ni | 0.05 | 0.07-0.37 | 0.08-0.10 | ND-0.27 | 0.17-0.33 | 0.05-0.17 | ND-0.30 | 0.16-0.21 | ND-0.34 | 0 ND-.62 |
| Pb | 0.05 | 0.09-0.46 | 0.09-0.14 | ND-0.06 | ND-0.08 | ND | ND-0.06 | ND | ND | ND-0.06 |
| Se | 0.25 | ND | ND | ND | ND | ND | ND | ND | ND | ND |
| Tl | 0.05 | ND-0.08 | ND | ND | ND | ND | ND | ND | ND | ND |
| Zn | 0.25 | 8.75-12.70 | 5.22-7.75 | 6.74-18.20 | 3.43-6.51 | 1.55-2.88 | 3.02-13.8 | 1.98-2.58 | 0.65-2.66 | 1.26-11.8 |
| Hg | 0.01 | 0.01-0.02 | 0.04-0.05 | 0.01-0.08 | 0.01-0.04 | 0.01-0.03 | 0.01-0.08 | 0.01-0.05 | 0.01-0.02 | 0.02-0.08 |
